# Test-retest reliability of the Online Elicitation of Personal Utility Functions (OPUF) approach for valuing the EQ-HWB-S

**DOI:** 10.1007/s10198-025-01769-4

**Published:** 2025-03-08

**Authors:** Aisha Moolla, Paul Schneider, Ole Marten, Clara Mukuria, Tessa Peasgood

**Affiliations:** 1 Sheffield Centre for Health and Related Research, University of Sheffield, 30 Regent Street, Sheffield, S1 4DA UK; 2 Department of Health Economics and Health Care Management, Bielefeld University, Bielefeld, Germany

**Keywords:** EQ-HWB, Health valuation, Multi-attribute value theory, Multi-criteria decision analysis, Personal utility function, Preference elicitation

## Abstract

**Introduction:**

The EQ Health and Wellbeing Short (EQ-HWB-S) is a new 9-item instrument designed to generate utility values. However, its length makes traditional preference elicitation challenging. The Online elicitation of Personal Utility Functions (OPUF) approach has been tested as a potential solution. This study aimed to assess the test-retest reliability of OPUF for valuing the EQ-HWB-S.

**Methods:**

The OPUF survey was administered twice, two weeks apart, to 220 German participants, including 73 from the general population and 147 patients with diabetes or rheumatic disease. Test-retest reliability was evaluated at individual and aggregate levels, examining dimension rankings, swing weights, level weights, and anchoring factors. Continuous data were analysed using the intraclass correlation coefficient (ICC), and ranking data were compared using Spearman’s correlation coefficient. Individual and aggregate level utility decrements were assessed using ICC and t-tests.

**Results:**

Approximately 36% of participants had significantly correlated dimension ranks, with 42% selecting the same top-ranked dimension. Poor agreement was shown in 70% of ICC values for individual dimension swing weights. For intermediate level weights, ICC values showed poor agreement in 70% and moderate agreement in 30% of responses. The kappa for individual pairwise comparison tasks was 0.64 (95% CI: 0.54–0.75) showing moderate agreement; however, the ICC for individual-level anchoring factors was 0.12 (*p* < 0.05), indicating poor agreement. Aggregate utility decrements across dimensions were similar.

**Conclusion:**

The OPUF approach generates reliable aggregate value sets for the EQ-HWB-S, but further exploration is needed to understand and address the reasons behind inconsistencies at the individual level.

**Supplementary Information:**

The online version contains supplementary material available at 10.1007/s10198-025-01769-4.

## Introduction

The EQ Health and Wellbeing (EQ-HWB) is a new instrument designed to measure health, social care, and carer-related quality of life [1]. The short version, the EQ-HWB-S, which has nine dimensions with five response levels, was specifically designed to derive utility values essential for economic evaluations of health and social care interventions. Dimensions include mobility (MO), daily activities (DA), exhaustion (EX), loneliness (LO), cognition (CG), anxiety (AX), sadness/depression (SD), control (CO), and physical pain (PA).

EQ-HWB utility values have been estimated using time trade-off (TTO) and discrete choice experiment (DCE) tasks [2] which are conventional decompositional preference elicitation techniques. However, the length and complexity of the EQ-HWB poses a challenge for these techniques [1, 2]. The EQ-HWB describes an extensive 1,953,125 health states, a tiny proportion of which are used in the estimation of the 36 utility decrements comprising the utility-algorithm. Conventional techniques necessitate large sample sizes to generate these decrements, and TTO and DCE tasks may impose cognitive burdens on participants, especially when contemplating nine dimensions simultaneously [1, 2, 3]. This challenge was evident during the development phase of the EQ-HWB-S, where the instrument was reduced to ≤ 10 items because the original 25-item version was not considered feasible for valuation using these methods [1, 2]. An alternative that can address these limitations is a compositional preference elicitation technique, the Personal Utility Functions (PUF) approach, that allows for the direct elicitation of partial values [4, 5]. The PUF approach allows estimation of utility functions for individuals and at the aggregate level and due to the approach, could be used to generate a utility function using a very small sample (*n* = 1) [4]. Other longer instruments such as the Health Utilities Index (HUI) [6], 15D [7], and Assessment of Quality of Life (AQoL) [8] have used semi-compositional techniques in which a combination of steps are used to generate utility values. This enables utility values to be generated at the aggregate level for each dimension as well as for the overall measure. This valuation approach has been shown to be feasible with evidence of validity of the utility scored measure [7, 9, 10].

An online PUF (OPUF) approach for EQ-HWB was recently tested in a UK and German population. The UK sample consisted of a general population sample and the German sample consisted of a general population sample and two patient samples (diabetes and rheumatic disease) [11]. It was shown that the OPUF method can generate value sets that align closely with those derived from more traditional methods, such as TTO and DCE, suggesting it is capable of producing reliable and meaningful preference data. In another UK-based study, OPUF-derived value sets for EQ-5D-5L demonstrated a prediction accuracy of 78% for hold-out DCE tasks, further validating its utility in preference elicitation [12]. Similarly, the method has shown feasibility in producing personal EQ-5D-5L value sets with high precision at the group level, even in small samples, indicating that it is a valid tool for eliciting health state preferences across different populations [5]. However, the reliability of the OPUF has not been assessed to date. Given the increasing uptake of the OPUF in eliciting utility values [5, 13, 14], it is crucial to determine whether the technique produces consistent results. This study aimed to evaluate the test-retest reliability of the OPUF method in valuing the EQ-HWB-S in the German sample.

## Materials and methods

### Sample

Adult participants were recruited from Germany using an online panel provided by a market research company, Bilendi, for participation in a validation study [11]. To compensate them for their time and ensure adequate engagement, participants received a monetary incentive of €7.50. The initial test was completed between 6 and 11 March 2023, and the retest was completed between 20 and 30 March 2023. Participants were re-invited for the retest phase through Bilendi, with each participant assigned a unique ID to ensure their responses could be accurately matched between the test and retest phases. Data were collected from a total of 330 participants. Of these participants, 110 were representative of the general population in terms of age and gender (which we refer to as the GP-sample), 110 were patients with diabetes (DM-sample), and 110 were patients with rheumatic disease (RA-sample). The same 330 participants were then invited to complete the survey again after 2 weeks. The participant preferences towards different health and wellbeing states were expected to remain consistent during this timeframe. To explore potential subgroup-specific differences, the sample was split and results were reported separately, allowing for a more nuanced analysis of how various factors may influence the findings.

### Preference elicitation survey

The OPUF employs a three-step valuation process to derive utility decrements for each dimension-level [5]. The first step aims to obtain swing weights for each dimension based on its relative importance. The next step aims to generate the level weights for each intermediate level of each dimension, which are anchored at the worst and best level. The final step aims to generate an anchoring factor that maps all health states on the quality-adjusted life year (QALY) scale, which is anchored at full health (100) or the dead state (0). These valuation steps are broken down and information is generated via a survey.

The EQ-HWB-S OPUF survey was delivered through an open-source online survey platform. The survey included questions related to the EQ-HWB-S and demographic questions (https://valorem.health/eqen-demo*).* The survey structured was as follows:

1. An introduction to the study and informed consent.
2. A warmup task in which participants reported their own EQ-HWB state and an adapted version of the EQ-VAS.
3. A dimension ranking task in which each of the level 5 EQ-HWB dimensions were ranked ‘from worst (first) to least bad (last)’ in a list format.
4. The dimension swing weights (between 0 and 100) were then elicited. The participant provided swing weights by indicating the value assigned to moving from the worst to the best level in each dimension. Moving from worst to the best level in the top ranked dimension from the previous task was given a fixed score of 100 and improvements in the other eight dimensions were scored relative to this improvement.
5. Intermediate levels of each dimension were assigned a weight (between 0 and 100) by asking participants to rate the intermediate levels. The best and worst levels were anchored at 100 and 0, respectively. When more severe levels were assigned higher weights than less severe levels, the response was considered illogical.
6. A pairwise comparison between the worst state (‘555555555’) and dead state was performed to elicit which scenario the participant preferred.
7. Anchoring was performed by assigning a value to the preferred state from the previous task. This was done by having participants indicate where the preferred state lies on a scale from 0 (representing the less preferred state in the previous task) and 100 (representing no health or wellbeing problems). Anchoring values were censored at -1.
8. Finally, participants provided demographic data and overall feedback.

The survey design for this study closely follows the methodology outlined in Schneider et al. [5, 11], with amendments made to accommodate the nine dimensions of the EQ-HWB-S and its respective level descriptions. While the core tasks—such as the warm-up task, dimension ranking, dimension swing weighting, and anchoring procedures—remain consistent with Schneider et al., our study utilized the EQ-HWB-S, which incorporates nine dimensions rather than five, and includes distinct level descriptions for each dimension. These modifications allow the method to capture preferences across a broader range of health states while staying aligned with the principles of the original framework.

### Data analysis

#### Estimating the utility decrements

**Fig. 1:**
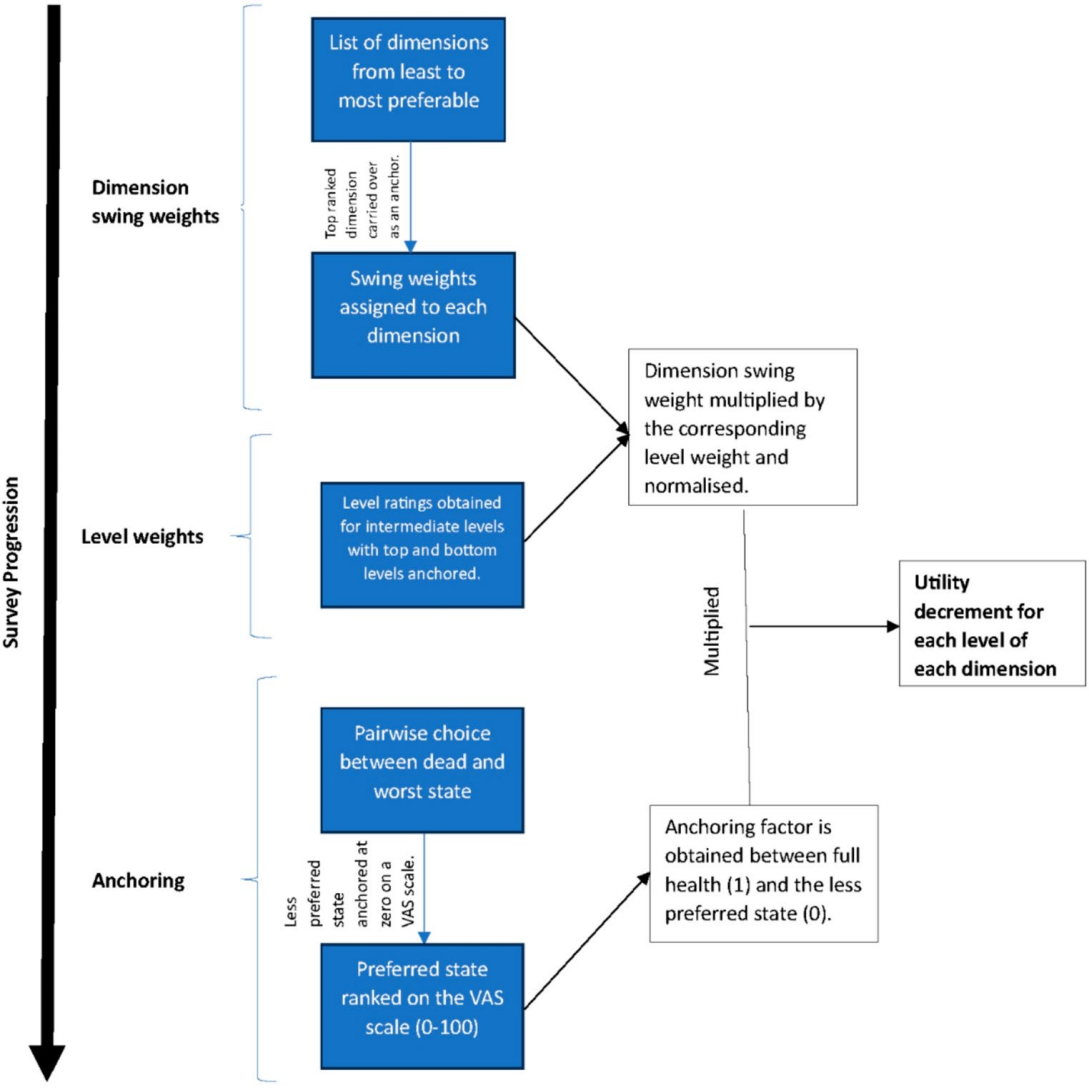
Survey tasks with corresponding outputs used to derive utility decrements

Figure 1. shows that components of the tasks and the process of deriving utility decrements. Utility decrements were estimated by multiplying the level rating by the corresponding dimension weight, the product of which was then normalised between 0 (best) and 1 (worst). An anchoring factor was estimated based on the position of the dead or worst health state (depending on the choice made by the participant) on a scale between full health and the worst state or dead. The normalised value was multiplied by the anchoring factor to produce a utility decrement, which was subtracted from 1 to produce a utility value. An additive model was used to derive the utility value of each health state for each participant as well as the entire population.

#### Test-retest

Test-retest reliability was assessed at both the aggregate and respondent level for the total sample population (GP + RA + DM samples), the general population (GP) sample, and the combined patient sample (RA + DM samples). Illogical responses were identified when participants selected zero-weights or weights of 100 for dimension swing weights or when severe levels were rated lower than less severe levels. Participants were excluded when they had more than two illogical responses. They were also excluded if they indicated indifference between full health and either death or state 555,555,555 in the anchoring task as meaningful value functions could not be derived from this.

General respondent characteristics, such as demographics, completion times, and the number of illogical responses, were summarised for both test and retest samples.

##### Dimension ranks

At the aggregate level, consistency was assessed by examining the proportion of participants who gave the same dimension the top ranking in both tests, reported as percentage agreement. The top ranked dimension acts as an anchor in the subsequent question, making this selection vital to the overall ranking of dimensions. A percentage agreement of ≥ 70% was considered adequate agreement between test and retest [15].

For individual-level dimension rankings, Spearman’s rank correlation coefficient was calculated for each participant as the sample size was too small to construct accurate confidence intervals around the weighted kappa statistic [16]. The size of the correlation was interpreted based on the following rho thresholds: negligible = 0.00-0.30, low = 0.30–0.50, moderate = 0.50 − 0.70, high = 0.70–0.90, and very high = 0.90-1.00 [17]. The proportion of participants who had significant positive correlations with a rho greater than 0.30 were reported.

##### Dimension swing weights

For individual-level dimension weights, we assessed reliability using the two-way mixed effects intraclass correlation coefficient (ICC) as both the degree of correlation and the agreement are relevant. A mixed effects model was selected as these tests (the test and retest) were the only “raters” of interest. There was no need to generalise inferences to other tests [17]. The ICC strength of agreement was classified as follows: poor = < 0.40, moderate = 0.41–0.59, good = 0.60–0.74, and excellent > 0.75 [18].

##### Level ratings

The reliability of intermediate level ratings was compared between the test and retest (the top and bottom levels are used as anchoring points and set to 0 and 100, respectively). Individual-level rating weights were then compared using the ICC as described above. Respondents with any illogical level ratings (e.g., more severe levels were rated better than less severe levels) were excluded from this part of the analysis as participants with illogical responses were thought to have interpreted the question incorrectly and the test aimed to evaluate consistency rather than understanding.

##### Anchoring factor

In the first step of the anchoring task, participants were asked to choose between the worst state ‘555555555’ (Scenario A) and ‘being dead’ (Scenario B). The consistency of this task was compared using percentage agreement. The agreement in this test was also assessed using the unweighted kappa statistic. The following cutoffs were used: poor = 0-0.2, fair = 0.21–0.40, moderate = 0.41–0.60, strong = 0.61–0.80, and near complete = > 0.81 [19]. In order to include all participants regardless of their selection (Scenario A or the Dead state) the ICC was calculated for the anchoring factor rather than the score produced using the visual analogue scale. The ICC was estimated for the overall group and for those who prefer death or the worst state separately. Utility values were censored at -1 for this analysis.

##### Utility decrements and value set

Both individual- and aggregate-level utility decrements were assessed for reliability between the test and retest. The value set, based on aggregate utility decrements, for both the test and retest were produced. The aggregate means of the decrements were compared using a paired t-test. However, the significance of the t-test is driven by the between-individual variances of the test and retest values, which may lead to an inaccurate result in the case of high variances [20]. As such, the empirical distributions of the aggregate level decrements were also compared using a Q-Q plot to perform a visual comparison and a two-sample Kolmogorov-Smirnov test to ascertain statistical significance. This test compares the cumulative distribution functions of each test and is based on both the location and shape of the distributions [21, 22]. Analysis of the distribution is also critical to understand due to its incorporation into economic analyses.

Individual-level decrements were compared using the ICC. Individual-level rankings of all health states scored based on the individual’s utility decrements were also compared using Spearman’s rank correlation decrements.

#### Impact of other factors on test-retest

Linear regression was performed to assess whether age (18–49, 50–64, 65+), gender, or sample group (general population vs. patient) predicted the cumulative difference in individual-level utility decrement values between test and retest. Two separate regressions were estimated, one with the main effects and the other including interaction terms between age and sample as patients were more likely to be older and the impact of age may differ in the two groups. The regressions aimed to assess whether these characteristics had an impact on the values. The cumulative difference was calculated as the sum of the absolute differences across all utility decrements. This metric was selected due to the very small values attached to the differences in utility decrements.

In all tests, p-values were considered significant at < 0.05. Normally distributed data were presented as the mean and standard deviation (SD) and non-normally distributed data were presented as the median and interquartile range (IQR). All statistical analyses were carried out using R version 4.3.1. This study was approved by the University of Bielefeld (ID: 2022 − 246) and all participants provided informed consent to participate in this study. During the preparation of this work the authors used ChatGPT 3.5 in order to improve the readability of the manuscript. After using this tool, the authors reviewed and edited the content as needed and take full responsibility for the content of the publication.

## Results

### Sample characteristics

In total, 330 and 257 participants completed the initial and retest surveys respectively. There were 110 participants in each sample (GP, DM, and RA) (Table 1). After the exclusion of participants, with illogical responses (*n* = 21 and 18, respectively) or unusable responses because they could not be matched to test responses (*n* = 19), the final analysis sample was 220 (66.67%) participants. Table 1 describes the demographic and test characteristics of all participants included in the analysis. In the GP group, the majority of participants were older than 50 years (52.1%), with the largest age category being 50–64 years (30.1%). In comparison, the majority of participants in the patient sample (83.0%) were also older than 50 years, but the largest age category was 65 + years (65.3%), suggesting that the patient group was older overall. Regarding education, most participants in both the GP (45.2%) and patient (59.2%) samples reported a medium level of education. Notably, the proportion of participants with a lower education level was slightly higher in the GP sample (15.1%) than in the patient sample (12.9%), while those with a higher education level were more represented in the GP sample (37.0%) compared to the patient sample (26.5%). Overall, there was a greater spread of education levels in the GP sample. Gender was fairly evenly split in both samples.

As anticipated, the median score for self-reported health was lower in the patient sample (69) compared to the GP sample (80). Mean survey completion times also varied between groups, with the GP sample completing the survey faster (11.4 min) than the patient sample (13.5 min).Reported gender demographics remained consistent, while education level and age varied between the test and retest. In the total sample, 4 participants reported a different age category and 24 reported a different education level. Completion rates and self-reported health when using a visual analogue scale (VAS) remained similar in all groups.

**Table 1:** Sample characteristics for each sub-group in the test and retest

	Test			Retest		
	Total population	GP sample	Patient sample	Total population	GP sample	Patient sample
Total sample	330	110	220	257	85	172
Excluded	21	0	21	18	5	13
Total included	309	110	199	239	80	159
Unmatched responses				19	7	12
Matched sample				220	73	147
**Age n (%)**						
18–29	17 (7.7%)	12 (16.4%)	5 (3.4%)	17 (7.7%)	12 (16.4%)	5 (3.4%)
30–39	22 (10%)	12 (16.4%)	10 (6.8%)	22 (10%)^a^	12 (16.4%)	10 (6.8%)^a^
40–49	20 (9.1%)	11 (15.1%)	9 (6.1%)	21 (9.6%)^a^	11 (15.1%)	10 (6.8%)^a^
50–64	48 (21.8%)	22 (30.1%)	26 (17.7%)	48 (21.8%)^a^	22 (30.1%)	26 (17.7%)^a^
65+	113 (51.4%)	16 (21.9%)	97 (66.0%)	112 (51.0%)^a^	16 (21.9%)	96 (65.3%)^a^
**Gender n (%)**						
Female	101 (45.9%)	31 (42.5%)	70 (47.6%)	101 (45.9%)	31 (42.5%)	70 (47.6%)
Male	118 (53.6%)	41 (56.2%)	77 (52.4%)	118 (53.6%)	41 (56.2%)	77 (52.4%)
Other	1 (0.5%)	1 (1.4%)	0 (0%)	1 (0.5%)	1 (1.4%)	0 (0%)
**Education n (%)**						
High	63 (28.6%)	26 (35.6%)	37 (25.2%)	66 (30.0%)^a^	27 (37.0%)^a^	39 (26.5%)^a^
Medium	132 (60.0%)	38 (52.1%)	94 (63.9%)	120 (54.5%)^a^	33 (45.2%)^a^	87 (59.2%)^a^
Low	22 (10.0%)	8 (11.0%)	14 (9.5%)	30 (13.6%)^a^	11 (15.1%)^a^	19 (12.9%)^a^
Not indicated	3 (1.4%)	1 (1.4%)	2 (1.4%)	4 (1.8%)^a^	2 (2.7%)^a^	2 (1.4%)
**Self-reported health**						
VAS score median (IQR)	70 (50–80)	75 (60–85)	69 (41–80)	70 (50–81)	80 (65–86)	69 (41–80)
**Survey completion times**						
Completion time (min) median (IQR)	14.2 (10.4–20.0)	11.1 (8-16.1)	15.8 (11.6–22.1)	13.0 (9.4–19.0)	11.4 (7.5–17.3)^a^	13.5 (10.4–20.0)

^a^indicates differences in test and retest

### Dimension ranks and swing weights

At the aggregate level, all samples had low consistency between test and retest for the top-ranked dimension. In the total sample, 93 of the 220 (42.27%) participants chose the same top ranked dimension. In the GP and patient samples, 36/73 and 57/147 participants, respectively, had consistently chosen the same top ranked dimension. This resulted in a percentage agreement of 49.32% and 38.78%, which was considered low. Similarly, consistency in individual-level dimension rankings was low, with only 36.46% of participants in the total sample, 41.10% of participants in the GP sample, and 34.01% of participants in the patient sample having significant positive correlations between tests.

For the swing weights, the individual-level ICC for five dimensions (MO, EX, LO, CO, and PA) were classified as having poor agreement; while four were considered moderate (DA, CG, AX, and SD) in the total sample (Table 2). In the GP sample, the ICC strength of agreement was classified as poor for three dimensions (MO, AX, and CO); moderate for five dimensions (DA, EX, LO, SD, and PA); and good for one dimension (CG). In the patient sample, there was poor agreement in all except one (AX) dimension, which had moderate agreement. Although there were similar levels of agreement between GP and patient samples when selecting the same top ranked dimension, dimension weights differed significantly. The patient sample had poor agreement in 8 out of 9 dimensions (89%), while the GP sample only demonstrated poor agreement in 3 of 9 dimensions (33%). Standard errors (SEs) are shown in Online Resource 1.

**Table 2:**
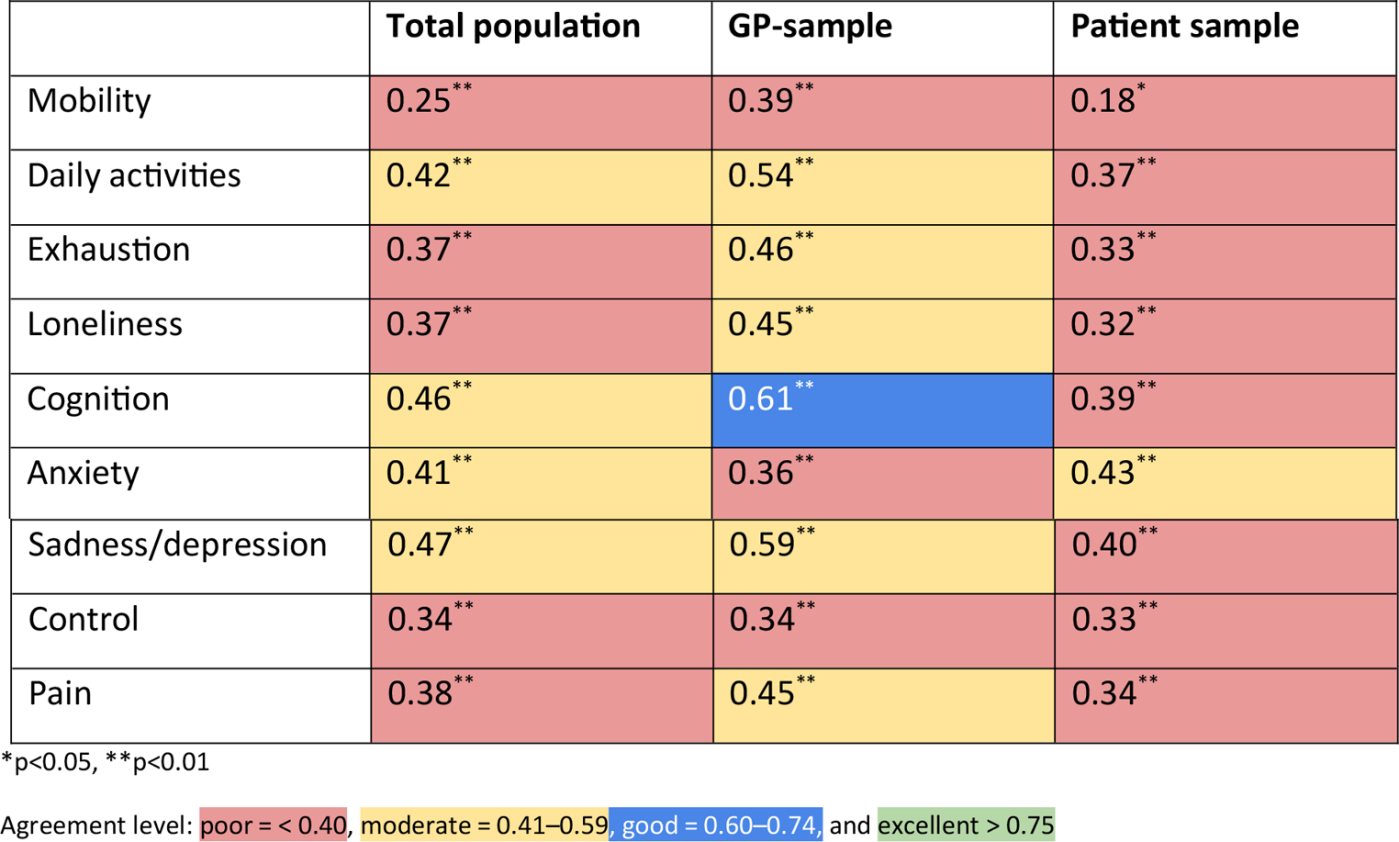
ICC values comparing dimension weights by sample

### Level ratings

In the total population sample, 45 (20.45%) and 46 (20.90%) of participants in the test and retest, respectively, produced illogical responses and were excluded for this part of the analysis. In total, 152 participants were included in the analysis, with 23 participants consistently producing illogical responses in both tests. The demographics of these participants are shown in Table 3. This indicates that illogical responses are being made consistently by those who are older and who have a medium level of education. ICC values analysing level weights were statistically significant (Table 4). These values showed poor agreement in 70.37% and moderate agreement in 29.63% of the level ratings.

**Table 3:** Demographics of participants with illogical response in the test and retest

	Demographics (*n* = 23)
**Age n (%)**	
18–29	1 (4.35%)
30–39	2 (8.70%)
40–49	1 (4.35%)
50–64	5 (21.74%)
65+	14 (60.87%)
**Gender n (%)**	
Female	12 (52.17%)
Male	11 (47.83%)
**Education n (%)**	
High	5 (21.74%)
Medium	14 (60.87%)
Low	5 (21.74%)

**Table 4:**
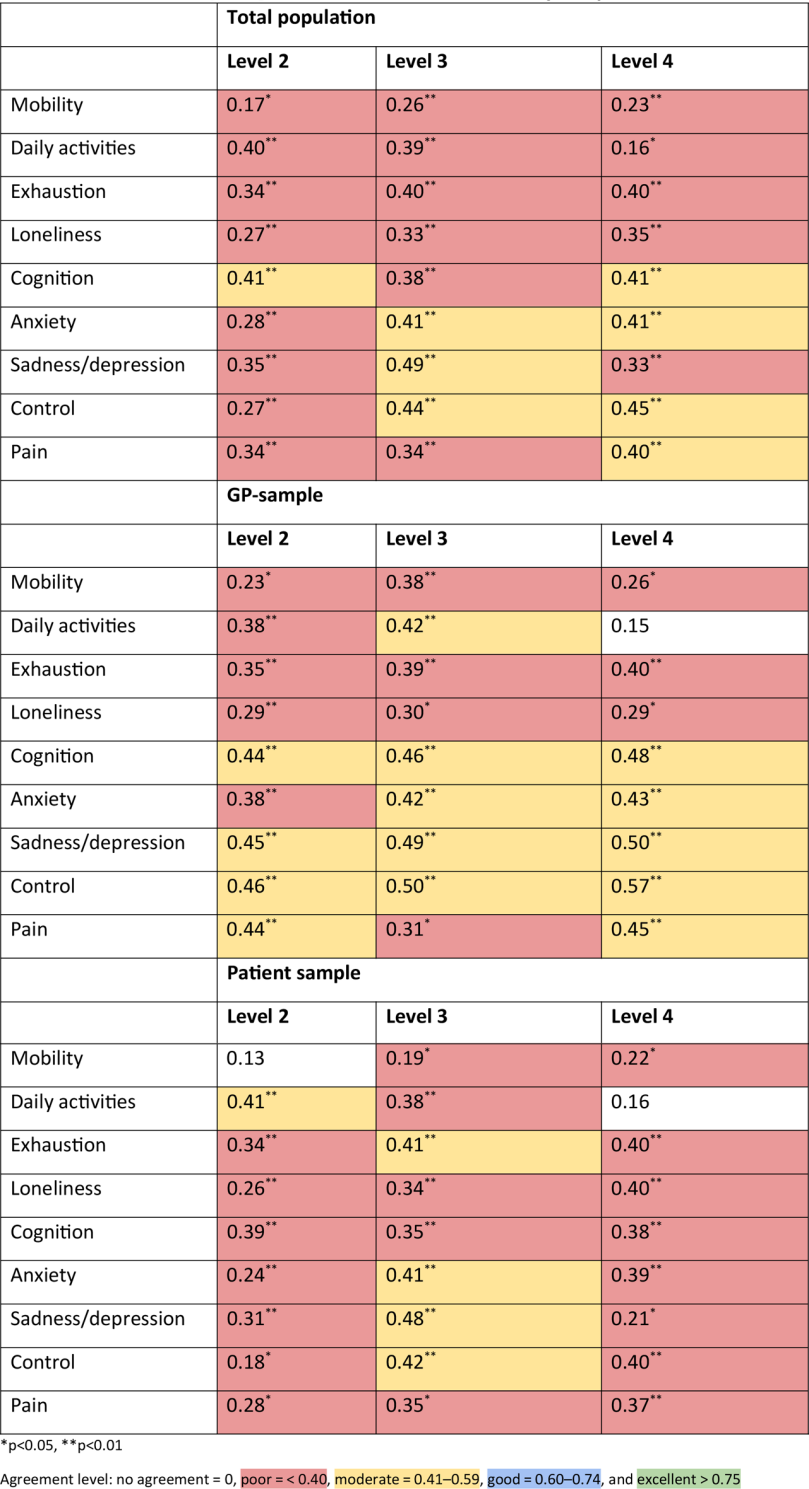
ICC values comparing absolute intermediate levels by sample

In the GP sample, 12 (16.44%) and 13 (17.81%) participants were excluded in the test and retest, respectively, due to illogical responses. In total, 54 participants were included in the final analysis, with 6 participants consistently producing illogical responses. All except one (UA level 4) ICC value, were statistically significant. Of those that were significant, more than half (54%) showed moderate agreement and the rest (46%) showed poor agreement.

In the patient sample, 33 (22.45%) participants, a substantially greater proportion of participants, were excluded in both the test and retest, respectively, due to illogical responses. In total, 114 participants were included in the final analysis, with 17 participants consistently producing illogical responses. Two ICC values were not statistically significant and showed no agreement between test and retest values. Of those that were significant, most (77%) showed poor agreement and the rest (23%) showed moderate agreement, illustrating poorer reliability in the patient sample compared to the GP sample. SEs are shown in Online Resource 2.

### Anchoring

In the total sample, the percentage agreement for the pairwise comparison task was 82.73%, indicating a high level of consistency. The unweighted kappa also showed good agreement, with a value of 0.64 (95% CI: 0.54–0.75). Overall, 117 participants consistently preferred the dead state, and 69 participants consistently preferred the worst health state. Only 34 participants changed their responses between the test and retest. In the total sample, the mean anchoring factors in the test and retest were − 0.09 and − 0.14, respectively. The overall ICC when comparing anchoring factors was 0.12 (confidence interval: -0.015-0.25), indicating poor agreement. When considering only those who consistently selected the dead state or the worst health state, the ICC was 0.12 (confidence interval: -0.057-0.30) and 0.12 (confidence interval: -0.12-0.34), indicating poor agreement.

The percentage agreement in the GP and patient samples for the pairwise comparison task was 83.56% and 82.31%, respectively, which was considered good agreement. Similarly, the unweighted kappas were 0.65 (95% CI: 0.48–0.83) and 0.64 (95% CI: 0.52–0.76), indicating good agreement. In the GP sample, 39 participants consistently preferred the dead state, and 21 participants consistently preferred the worst health state, with 13 participants changing their responses between the test and retest. In the patient sample, 73 participants consistently preferred the dead state, and 48 participants consistently preferred the worst health state, with 26 participants changing their responses.

The mean anchoring factors were − 0.13 and − 0.08 in the test for the GP and patient samples, respectively. The mean retest anchoring factors were − 0.14 and − 0.14 in the GP and patient samples, respectively. The ICC produced when comparing anchoring factors in the overall group was − 0.00066 (*p* > 0.05) and 0.16 (*p* < 0.05), indicating no agreement and poor agreement in the GP and patient samples. Among those in the GP sample who consistently chose the dead state, the ICC was − 0.017 (*p* > 0.05), and among those who consistently chose the worst health state, the ICC was 0.57 (*p* < 0.01). This indicates that those who select the worst state produce a more consistent anchoring value than those who preferred the dead state. Among those in the patient sample who consistently chose the dead state, the ICC was 0.19 (*p* > 0.05), and among those who consistently chose the worst health state, the ICC was − 0.040 (*p* > 0.05). This indicates no agreement in either group.

### Utility decrements and value set

Table 5 shows the ICC values produced when comparing the 36 individual level utility decrements. In the overall sample, 35 of the 36 ICC values were significant. Of those, the ICC showed poor agreement in 23 decrements and moderate agreement in 12 decrements.

**Table 5:**
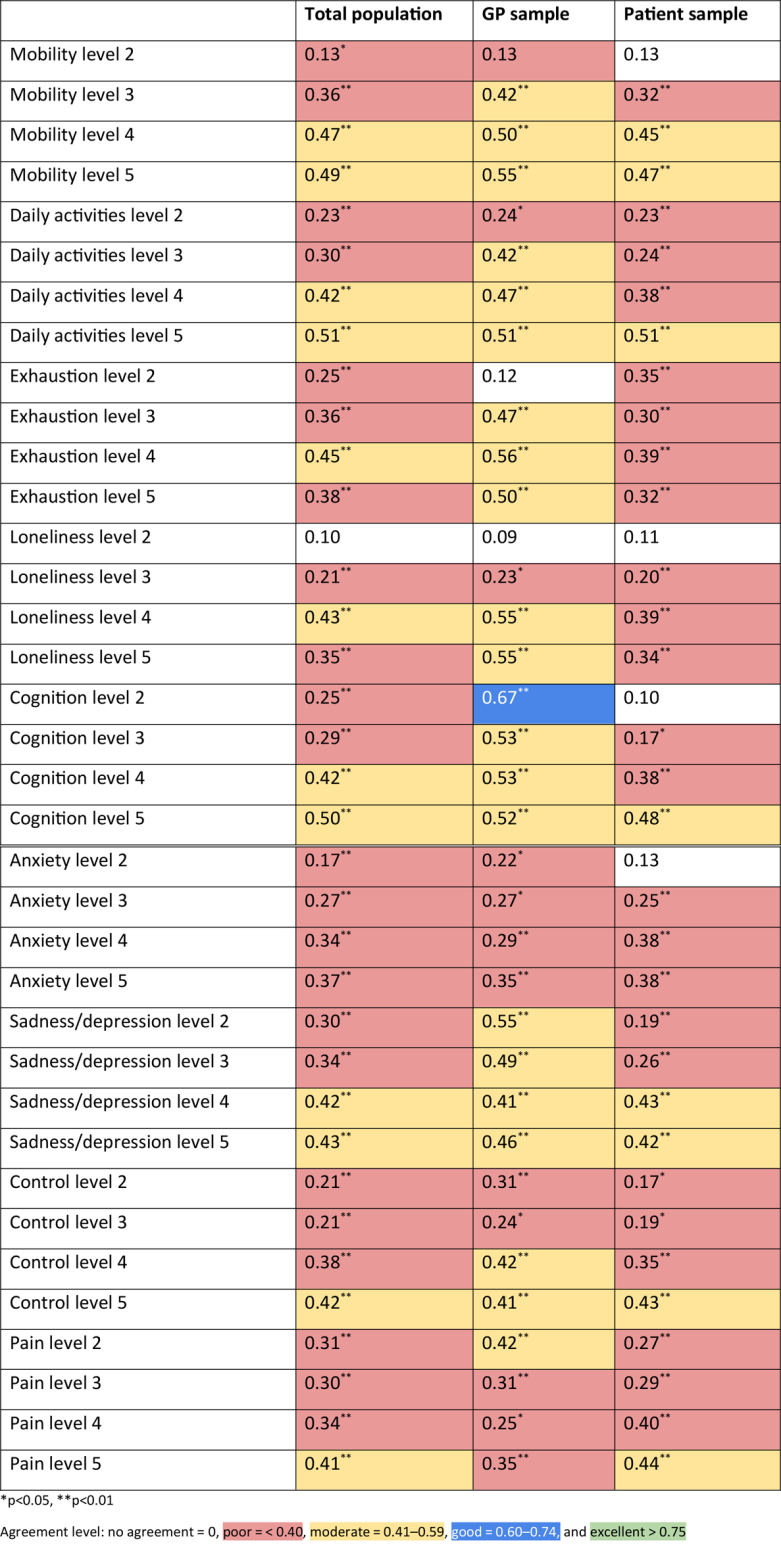
ICC values when comparing individual level utility decrements

In the GP sample, the ICC values were significant in 33 of the decrements. Of those that were significant, 1 ICC value indicated good agreement (CG2), 21 indicated moderate agreement, and 11 indicated poor agreement. In the patient sample, the ICC values were significant in 32 of the decrements. Of those that were significant, 8 indicated moderate agreement and 24 indicated poor agreement, indicating lower reliability in the patient sample compared to the GP sample. SEs are shown in Online Resource 3.

**Fig. 2:**
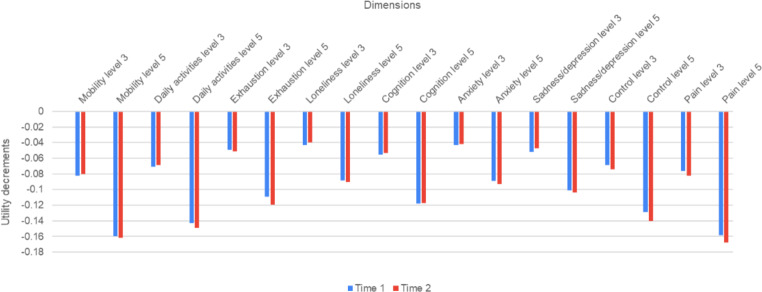
Aggregate level utility decrements for levels three and five in the test and retest (total sample)

**Fig. 3:**
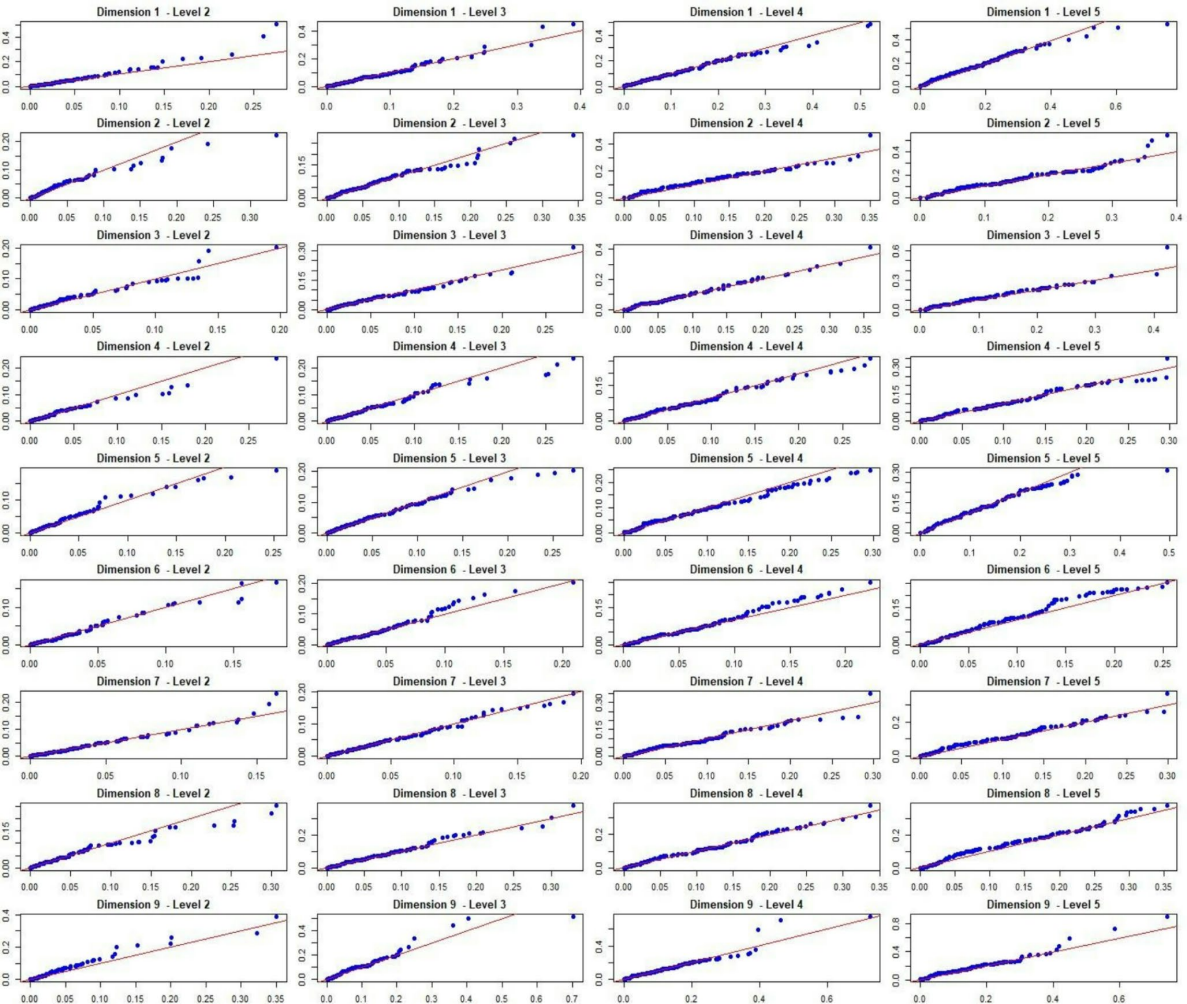
Q-Q plots based on the empirical distributions of the aggregate level utility decrements in the test and retest (Total sample). Dimensions: 1, Mobility; 2, Daily activities; 3, Exhaustion; 4, Loneliness; 5, Cognition; 6, Anxiety; 7, Sadness/depression; 8, Control; 9, Pain

The aggregate level utility decrements were also compared between the test and retest. The mean overall utility decrement was similar (0.08) in both the test and retest. Figure 2 shows the small absolute differences in aggregate level decrements between the test and retest in each dimension for the total sample. The mean absolute difference was 0.004. Figure 3 provides a graphical representation of the distributions of the utility decrements. The Q-Q plots (Fig. 3) show that the distributions of the aggregate utility decrements are similar between the test and retest, with many plots appearing to intercept at zero and lie on the 45 degree line.

Table 6 shows the Kolmogorov-Smirnov test and a paired t-test results from the comparison of aggregate level utility decrements. In the total, GP, and patient samples, the D and t statistics are not statistically significant with the exception of the D-statistic for EX5 in the total sample and PA3 in the patient sample, indicating that test and retest distribution of these decrements were not significantly different. The t-statistic was not statistically significant for SD3 in the GP sample, indicating differences in means. This may not be evident in the KS test given that this test is partly driven by the distribution, giving the mean less influence over the overall significance of the test.

**Table 6:** Kolmogorov-Smirnov and t-test results by sample

	Total population		GP sample		Patient sample	
	D_*n*_ (*p*-value)	t (*p*-value)	D_*n*_ (*p*-value)	t (*p*-value)	D_*n*_ (*p*-value)	t (*p*-value)
MO2	0.06 (0.84)	-0.63 (0.52)	0.10 (0.89)	0.69 (0.49)	0.06 (0.95)	-1.15 (0.25)
MO3	0.07 (0.60)	0.35 (0.73)	0.12 (0.64)	0.96 (0.34)	0.06 (0.95)	-0.23 (0.82)
MO4	0.05 (0.95)	0.54 (0.59)	0.07 (0.20)	-0.002 (1.00)	0.05 (1.00)	0.66 (0.51)
MO5	0.08 (0.53)	-0.31 (0.76)	0.11 (0.78)	0.004 (1.00)	0.07 (0.89)	-0.36 (0.72)
DA2	0.08 (0.45)	0.67 (0.50)	0.12 (0.64)	1.19 (0.24)	0.09 (0.61)	-0.02 (0.98)
DA3	0.05 (0.95)	0.45 (0.65)	0.08 (0.97)	0.94 (0.35)	0.07 (0.80)	-0.07 (0.94)
DA4	0.08 (0.45)	-0.85 (0.39)	0.11 (0.78)	0.25 (0.80)	0.11 (0.35)	-1.24 (0.22)
DA5	0.08 (0.53)	-0.86 (0.39)	0.10 (0.89)	0.29 (0.77)	0.12 (0.28)	-1.27 (0.21)
EX2	0.07 (0.61)	-1.06 (0.29)	0.11 (0.78)	-1.08 (0.28)	0.10 (0.52)	-0.41 (0.69)
EX3	0.10 (0.18)	-0.64 (0.52)	0.15 (0.38)	-0.83 (0.41)	0.09 (0.61)	-0.22 (0.82)
EX4	0.05 (0.95)	-0.49 (0.62)	0.14 (0.50)	-0.13 (0.89)	0.07 (0.81)	-0.49 (0.62)
EX5	0.13 (0.04)^*^	-1.75 (0.08)	0.18 (0.20)	-1.34 (0.19)	0.13 (0.17)	-1.25 (0.21)
LO2	0.06 (0.84)	0.67 (0.50)	0.10 (0.89)	0.42 (0.67)	0.06 (0.95)	0.52 (0.61)
LO3	0.05 (0.95)	0.67 (0.50)	0.11 (0.78)	0.32 (0.75)	0.06 (0.95)	0.59 (0.56)
LO4	0.05 (0.95)	0.31 (0.76)	0.12 (0.64)	0.06 (0.96)	0.07 (0.89)	0.32 (0.75)
LO5	0.08 (0.45)	-0.44 (0.66)	0.11 (0.78)	-0.77 (0.44)	0.09 (0.61)	0.03 (0.97)
CG2	0.07 (0.69)	-0.34 (0.74)	0.12 (0.64)	0.14 (0.89)	0.07 (0.80)	-0.46 (0.65)
CG3	0.06 (0.84)	0.64 (0.52)	0.10 (0.89)	0.71 (0.48)	0.04 (1.00)	0.35 (0.73)
CG4	0.07 (0.69)	0.69 (0.49)	0.15 (0.38)	-0.02 (0.98)	0.08 (0.71)	0.80 (0.43)
CG5	0.08 (0.45)	0.27 (0.78)	0.14 (0.50)	-0.28 (0.78)	0.07 (0.80)	0.48 (0.63)
AX2	0.07 (0.69)	0.20 (0.85)	0.10 (0.89)	-0.30 (0.77)	0.10 (0.43)	0.49 (0.63)
AX3	0.06 (0.84)	0.16 (0.88)	0.08 (0.97)	0.71 (0.48)	0.05 (0.98)	-0.45 (0.65)
AX4	0.05 (0.98)	0.38 (0.70)	0.11 (0.78)	1.42 (0.16)	0.05 (0.98)	-0.90 (0.37)
AX5	0.10 (0.27)	-0.69 (0.49)	0.08 (0.97)	0.87 (0.39)	0.11 (0.35)	-1.67 (0.10)
SD2	0.06 (0.76)	0.74 (0.46)	0.10 (0.89)	1.92 (0.06)	0.07 (0.89)	-0.15 (0.88)
SD3	0.06 (0.76)	1.46 (0.15)	0.11 (0.78)	2.23 (0.03)^*^	0.05 (1.00)	0.31 (0.76)
SD4	0.08 (0.53)	1.02 (0.31)	0.15 (0.38)	1.23 (0.22)	0.09 (0.61)	0.35 (0.73)
SD5	0.09 (0.38)	-0.63 (0.53)	0.10 (0.89)	0.56 (0.58)	0.12 (0.28)	-1.14 (0.25)
CO2	0.10 (0.84)	0.21 (0.84)	0.14 (0.50)	-0.31 (0.76)	0.05 (0.98)	0.44 (0.66)
CO3	0.07 (0.61)	-0.84 (0.40)	0.11 (0.78)	-0.55 (0.58)	0.08 (0.71)	-0.63 (0.53)
CO4	0.09 (0.38)	-0.97 (0.33)	0.12 (0.64)	-1.02 (0.31)	0.09 (0.61)	-0.44 (0.66)
CO5	0.12 (0.07)	-1.74 (0.08)	0.12 (0.64)	-0.58 (0.56)	0.13 (0.17)	-1.74 (0.08)
PA2	0.1 (0.22)	-1.18 (0.24)	0.10 (0.89)	0.43 (0.67)	0.13 (0.17)	-1.55 (0.12)
PA3	0.13 (0.06)	-0.91 (0.37)	0.11 (0.78)	-0.16 (0.88)	0.16 (0.04)^*^	-0.98 (0.33)
PA4	0.04 (0.99)	-0.39 (0.70)	0.10 (0.89)	-0.19 (0.84)	0.07 (0.80)	-0.35 (0.73)
PA5	0.08 (0.53)	-1.08 (0.28)	0.12 (0.64)	-0.16 (0.87)	0.11 (0.35)	-1.28 (0.20)

*p < 0.05, statistically significant

Dimensions: MO, Mobility; DA, Daily activities; EX, Exhaustion; LO, Loneliness; CG, Cognition; AX, Anxiety; SD, Sadness/depression; CO, Control; PA, Pain

The final health state rankings were compared between the test and retest using Spearman’s rank correlation test. The rho was 0.26 (*p* < 0.05), 0.26 (*p* < 0.05), and 0.26 (*p* < 0.05) in the total, GP, and patient samples, respectively, indicating a low to negligible positive monotonic relationship between the test and retest health state ranks.

### Regression analysis

The regression results showing the relationship between age, gender, and patient sample and the cumulative difference in utility decrement are shown in Online Resource 4. The age coefficients were positive indicating that those who were older had larger cumulative differences. However, only age 50–64 was statistically significant in Model 1 and this appeared to be associated with whether they were in the GP or patient sample (Model 2) as it was no longer statistically significant when the interaction term was introduced in the latter regression. The patient coefficients were negative in both regressions. Neither were statistically significant but the coefficient in Model 2 increased in size when the interaction term was introduced. Being male was associated with a lower difference and this was borderline statistically significant in Model 1 and statistically significant in Model 2. Overall, these findings suggest that while gender has some impact on the cumulative difference in utility decrement values, the effect for age was not uniform across different samples.

## Discussion

The findings of this study highlight several noteworthy aspects regarding the test-retest reliability of the OPUF EQ-HWB-S. There was a lack of consistency demonstrated in the separate tasks at the individual and aggregate level with a notable exception of the pairwise task in the anchoring step. This had an impact on the agreement of the utility values at the individual level which were not consistent, but this lack of consistency was not shown in the aggregate utility values.

When considering dimension rankings, only 42.27% of total participants chose the same top ranked dimension. This suggests a notable degree of variability in individual responses. The ICC values assessing dimension swing weights across all samples revealed predominantly moderate to poor agreement, indicating a lack of consistency in the ranking of health dimensions. While one dimension in the GP sample showed good agreement, the overall pattern suggests that participants struggled to maintain consistent responses over the test-retest period. The inconsistency observed in intermediate level weights further emphasises the challenges associated with individual-level responses, with ICC values consistently indicating moderate to poor agreement.

Interestingly, the kappa values (0.6) and percentage-agreement values (83%) derived from pairwise comparisons demonstrated good agreement across all samples, contradicting the inconsistency observed in preceding tasks. Although no studies have evaluated the reliability of the OPUF, conventional valuation methods have been assessed. The poor agreement observed in ICC values for anchoring factors underscores potential challenges in maintaining consistent reference points across test and retest sessions. This finding may be particularly relevant in understanding the impact of variations in participants’ comprehension or interpretation of the survey instructions.

Although no studies have assessed the reliability of OPUF valuation tasks, prior research has evaluated the reliability of similar methods like DCE and VAS. A German study estimated the test-retest reliability of DCE for the 10-item QLU-C10D, with a kappa of 0.605 and 80.2% agreement, similar to our findings [23]. Other studies report kappa values for DCE ranging from 0.49 to 0.78 across various populations and administration methods [24, 25, 26, 27]. These results show moderately good agreement across methods, suggesting that paired comparisons are generally consistent for participants. However, our study found participants struggled with ranking dimensions and assigning weights individually, indicating potential cognitive challenges that warrant further investigation.

In terms of VAS, a Spanish study reported an ICC of 0.90 [28], a British study 0.78 [29], and a Dutch study 0.94 [30], all indicating strong reliability. The differences in findings may stem from variations in how the scale is applied in OPUF, where the 0-100 scale is used inconsistently across tasks. Similarly, TTO tasks tend to show lower consistency (ICC of 0.84) compared to VAS [28, 30]. In our study, utility decrements showed moderate to poor individual-level consistency, except for one GP sample decrement. Aggregate-level utility decrements, however, exhibited high consistency between test and retest sessions. The lack of trade-off tasks in the OPUF and narrow utility value ranges likely contributed to the individual-level inconsistencies. The observed inconsistency in individual-level coefficients, contrasted with the more consistent utility values at the aggregate level, can be explained by the averaging effect in the OPUF method. While individual responses may show variability, these differences tend to cancel each other out at the aggregate level, resulting in more stable and consistent utility values across the sample.

While this evidence suggests these valuation methods are reliable, the results of the present study do not allow us to conclude whether the valuation approach or the instrument is driving the observed results. However, the digital nature of the survey may contribute to the challenges faced by participants. The steep learning curve and the attention-demanding nature of the tasks may have led to the high degree of inconsistency [31]. These issues may be likely to occur in those with poor digital literacy, suggesting the potential need to include interviewers [32]. Additionally, while the analysis initially excluded illogical responses, the relatively high rate of such responses raises important questions about the effectiveness of the OPUF method. This could suggest that the method may not be as intuitive or accessible for all participants, particularly those who are older and have a medium level of education.

Our regression analyses provide further context. Age was associated with cumulative differences in utility decrement values, with older participants generally exhibiting larger differences. However, this effect was not uniform across samples and was only statistically significant for the 50–64 age group in Model 1, where it appeared to be influenced by sample type (GP or patient). When controlling for the interaction between age and sample type in Model 2, the effect of age was no longer significant. Gender also showed some association, with being male linked to lower differences in cumulative utility decrement values, achieving statistical significance in Model 2. These findings suggest that individual-level inconsistencies may, in part, be influenced by demographic factors such as age and gender, although the effect sizes were modest.

Furthermore, for dimension weight, level ratings, and individual utility decrements, the patient sample showed poorer reliability than the general population sample. However, this difference was not apparent when controlling for age in the regression suggesting the poorer patient sample performance was in part driven by the higher mean age in the patient sample. Utility scores can, however, be influenced by the severity of illness, symptom burden, and the subjective experience of living with a chronic condition, which may lead patients to evaluate their health status differently than individuals in the general population [33, 34, 35, 36]. This phenomenon is often referred to as response shift, where individuals adjust their internal standards, values, or conceptualizations of health over time due to their illness experience [37]. Equally, some patients may have answered the questions in relation to their own health as they were experiencing it rather than treating the tasks as being related to hypothetical scenarios. This would result in changes in values even within a short time if symptoms and functioning varied. In contrast, while the general population may also respond based on their current health status [36], they are more likely to choose the same answer due to the complexity of the questions and, due to lack of real world experience of disease states, a lack of respondent ability, which is the capability to recall information from memory and incorporate it into verbally articulated judgments [38, 39]. These factors may lead participants in the general population to use decision heuristics [39]. As such, these participants are likely to provide consistent answers. Inconsistencies in participants’ responses also extended to demographic information. However, these discrepancies likely reflect inattentiveness during survey completion, which is a known limitation of online panels [40], rather than a change in respondents. Future research could address this issue by implementing additional measures, such as attention checks, to improve data reliability.

The identified challenges in individual-level responses emphasise the need for further qualitative research to explore which specific tasks participants find challenging and the underlying reasons for inconsistencies as participant understanding and engagement appear to be crucial aspects to improving results of the OPUF EQ-HWB-S. Insights gained from qualitative studies can inform refinements in the survey design, potentially enhancing participant understanding and reducing response variability. Once design adjustments are made based on qualitative findings, reassessment of the test-retest reliability of the instrument is essential. This iterative process of refinement and re-evaluation is crucial for ensuring the validity and reliability of valuation methods used to generate utility values for health-related quality of life assessments.

This study has some limitations. First, those with multiple illogical responses or indifference in anchoring were excluded from the study. This may lead to an overestimation in the agreement between tests. Second, OPUF relies on using VAS scales for some of the tasks which are known to have some biases, e.g., response spreading is a common phenomenon seen when using a VAS scale, which may have occurred in our study but could not be accounted for in the analysis [41]. Third, participants were recruited for online completion of the survey. This may have led to the inclusion of those with better digital literacy. However, efforts were made to ensure that participants were representative of the population. Fouth, a German sample was used for this study which may limit the generalisability of the study although the assessment focuses on the reliability of the valuation tasks rather than the results generated by OPUF. Finally, it is important to note that the reliability evaluated in this study differs from traditional reliability assessments used in other methods such as TTO and DCE. Given that the OPUF method allows for the examination of consistency at different levels for various tasks, it may not be directly comparable to these conventional methods. This distinction should be considered when interpreting and comparing the results to other studies.

The observed differences between the test and retest for individual and aggregate-level utility decrements raise questions about the applicability of the OPUF approach with a sample size of one [4]. While aggregate-level utility decrements demonstrated high similarity between test and retest, individual-level utility decrements exhibited poor to moderate agreement, indicating potential limitations in the survey’s ability to capture stable “personal” utility functions. Inconsistencies in individual-level tasks, which are intended to be simpler than other elicitation tasks, bring into question the validity of the final utility values. Further research is required to explore these concerns further. In particular, incorporating an element of respondent-feedback analysis could provide valuable insights into the validity and feasibility of tasks, especially in online environments, given the short completion times observed in this study. Such feedback may help identify sources of inconsistency, such as task complexity or interpretation issues, and could guide refinements to improve consistency in individual-level utility estimates. Additionally, an analysis of the minimum sample size required for the meaningful application of the OPUF approach is warranted. The analysis demonstrates the lack of reliability at an individual level, however, it is unclear whether this is driven by the OPUF approach, the complexity of the EQ-HWB-S, and/or the online nature of the tool. Thus, the reliability of the OPUF using different approaches such as self-completion with an observer or interviewer-administration of the OPUF is required in future. Understanding the trade-off between individual and aggregate-level reliability is vital for researchers and policymakers seeking to implement this approach in diverse contexts.

## Conclusion

In conclusion, while the OPUF EQ-HWB-S holds promise as a tool for generating utility values for health-related quality of life measures, this study illustrates that the OPUF approach produces reliable value sets for the EQ-HWB-S on the aggregate group level only. Individual level tasks still lack reliability when using this approach. This necessitates careful consideration and refinement of the OPUF method in order to produce consistent individual-level responses.

## Electronic supplementary material

Below is the link to the electronic supplementary material.

Supplementary Material 1
