# Supplementary material for: Test-retest reliability of the Online Elicitation of Personal Utility Functions (OPUF) approach for valuing the EQ-HWB-S
Source: Eur J Health Econ. 2025 Mar 8;26(7):1175–90. doi: 10.1007/s10198-025-01769-4 (PMC12431937; doi:10.1007/s10198-025-01769-4)
Supplement: Supplementary file 1 — Supplementary Material 1 [file 10198_2025_1769_MOESM1_ESM.docx]

**Test-retest reliability of the Online elicitation of Personal Utility Functions (OPUF) approach for valuing the EQ-HWB-S**

Aisha Moolla^a*^, Paul Schneider^a^, Ole Marten^b^, Clara Mukuria^a^, Tessa Peasgood^a^

^a^Sheffield Centre for Health and Related Research, University of Sheffield, Sheffield, UK

^b^Department of Health Economics and Health Care Management, Bielefeld University, Bielefeld, Germany

^*^Corresponding author:

E-mail address: amoolla1@sheffield.ac.uk

Full postal address: Sheffield Centre for Health and Related Research, University of Sheffield, 30 Regent Street, Sheffield, UK, S1 4DA

**Online Resource 1. Standard errors of ICC values comparing dimension weights by sample**

| **Dimension** | **ICC** | **Standard Error** |
| --- | --- | --- |
| **Total population** | | |
| Mobility | 0.25 | 0.06 |
| Daily activities | 0.42 | 0.06 |
| Exhaustion | 0.37 | 0.06 |
| Loneliness | 0.37 | 0.06 |
| Cognition | 0.46 | 0.05 |
| Anxiety | 0.41 | 0.06 |
| Sadness/depression | 0.47 | 0.05 |
| Control | 0.34 | 0.06 |
| Pain | 0.38 | 0.06 |
| **GP sample** | | |
| Mobility | 0.39 | 0.10 |
| Daily activities | 0.54 | 0.08 |
| Exhaustion | 0.46 | 0.09 |
| Loneliness | 0.45 | 0.09 |
| Cognition | 0.61 | 0.07 |
| Anxiety | 0.36 | 0.10 |
| Sadness/depression | 0.59 | 0.08 |
| Control | 0.34 | 0.10 |
| Pain | 0.45 | 0.09 |
| **Patient sample** | | |
| Mobility | 0.18 | 0.08 |
| Daily activities | 0.37 | 0.07 |
| Exhaustion | 0.33 | 0.07 |
| Loneliness | 0.32 | 0.07 |
| Cognition | 0.39 | 0.07 |
| Anxiety | 0.43 | 0.07 |
| Sadness/depression | 0.4 | 0.07 |
| Control | 0.33 | 0.07 |
| Pain | 0.34 | 0.07 |

**Online Resource 2. Standard errors of ICC values comparing absolute intermediate levels by sample**

| **Dimension** | **Level** | **ICC** | **Standard Error** |
| --- | --- | --- | --- |
| **Total population** | | | |
| Mobility | 2 | 0.17 | 0.08 |
| Mobility | 3 | 0.26 | 0.07 |
| Mobility | 4 | 0.23 | 0.08 |
| Daily activities | 2 | 0.4 | 0.07 |
| Daily activities | 3 | 0.39 | 0.07 |
| Daily activities | 4 | 0.16 | 0.08 |
| Exhaustion | 2 | 0.34 | 0.07 |
| Exhaustion | 3 | 0.4 | 0.07 |
| Exhaustion | 4 | 0.4 | 0.07 |
| Loneliness | 2 | 0.27 | 0.07 |
| Loneliness | 3 | 0.33 | 0.07 |
| Loneliness | 4 | 0.35 | 0.07 |
| Cognition | 2 | 0.41 | 0.07 |
| Cognition | 3 | 0.38 | 0.07 |
| Cognition | 4 | 0.41 | 0.07 |
| Anxiety | 2 | 0.28 | 0.07 |
| Anxiety | 3 | 0.41 | 0.07 |
| Anxiety | 4 | 0.41 | 0.07 |
| Sadness/depression | 2 | 0.35 | 0.07 |
| Sadness/depression | 3 | 0.49 | 0.06 |
| Sadness/depression | 4 | 0.33 | 0.07 |
| Control | 2 | 0.27 | 0.08 |
| Control | 3 | 0.44 | 0.07 |
| Control | 4 | 0.45 | 0.07 |
| Pain | 2 | 0.34 | 0.07 |
| Pain | 3 | 0.34 | 0.07 |
| Pain | 4 | 0.4 | 0.07 |
| **GP sample** | | | |
| Mobility | 2 | 0.23 | 0.13 |
| Mobility | 3 | 0.38 | 0.12 |
| Mobility | 4 | 0.26 | 0.13 |
| Daily Activities | 2 | 0.38 | 0.12 |
| Daily Activities | 3 | 0.42 | 0.11 |
| Daily Activities | 4 | 0.15 | 0.13 |
| Exhaustion | 2 | 0.35 | 0.12 |
| Exhaustion | 3 | 0.39 | 0.12 |
| Exhaustion | 4 | 0.4 | 0.11 |
| Loneliness | 2 | 0.29 | 0.12 |
| Loneliness | 3 | 0.3 | 0.13 |
| Loneliness | 4 | 0.29 | 0.13 |
| Cognition | 2 | 0.44 | 0.11 |
| Cognition | 3 | 0.46 | 0.11 |
| Cognition | 4 | 0.48 | 0.11 |
| Anxiety | 2 | 0.38 | 0.12 |
| Anxiety | 3 | 0.42 | 0.11 |
| Anxiety | 4 | 0.43 | 0.11 |
| Sadness/Depression | 2 | 0.45 | 0.11 |
| Sadness/Depression | 3 | 0.49 | 0.10 |
| Sadness/Depression | 4 | 0.5 | 0.10 |
| Control | 2 | 0.46 | 0.11 |
| Control | 3 | 0.5 | 0.10 |
| Control | 4 | 0.57 | 0.09 |
| Pain | 2 | 0.44 | 0.11 |
| Pain | 3 | 0.31 | 0.12 |
| Pain | 4 | 0.45 | 0.11 |
| **Patient sample** | | | |
| Mobility | 2 | 0.13 | 0.10 |
| Mobility | 3 | 0.19 | 0.10 |
| Mobility | 4 | 0.22 | 0.10 |
| Daily Activities | 2 | 0.41 | 0.08 |
| Daily Activities | 3 | 0.38 | 0.09 |
| Daily Activities | 4 | 0.16 | 0.10 |
| Exhaustion | 2 | 0.34 | 0.09 |
| Exhaustion | 3 | 0.41 | 0.08 |
| Exhaustion | 4 | 0.4 | 0.09 |
| Loneliness | 2 | 0.26 | 0.09 |
| Loneliness | 3 | 0.34 | 0.09 |
| Loneliness | 4 | 0.4 | 0.08 |
| Cognition | 2 | 0.39 | 0.09 |
| Cognition | 3 | 0.35 | 0.09 |
| Cognition | 4 | 0.38 | 0.09 |
| Anxiety | 2 | 0.24 | 0.09 |
| Anxiety | 3 | 0.41 | 0.08 |
| Anxiety | 4 | 0.39 | 0.09 |
| Sadness/Depression | 2 | 0.31 | 0.09 |
| Sadness/Depression | 3 | 0.48 | 0.08 |
| Sadness/Depression | 4 | 0.21 | 0.10 |
| Control | 2 | 0.18 | 0.10 |
| Control | 3 | 0.42 | 0.08 |
| Control | 4 | 0.4 | 0.09 |
| Pain | 2 | 0.28 | 0.09 |
| Pain | 3 | 0.35 | 0.09 |
| Pain | 4 | 0.37 | 0.09 |

**Online Resource 3. Standard errors of ICC values when comparing individual level utility decrements**

| **Dimension** | **Level** | **Coefficient (ICC3)** | **Standard Error** |
| --- | --- | --- | --- |
| **Total population** | | | |
| Mobility | 2 | 0.13 | 0.07 |
| Mobility | 3 | 0.36 | 0.06 |
| Mobility | 4 | 0.47 | 0.05 |
| Mobility | 5 | 0.49 | 0.05 |
| Daily activities | 2 | 0.23 | 0.06 |
| Daily activities | 3 | 0.3 | 0.06 |
| Daily activities | 4 | 0.42 | 0.06 |
| Daily activities | 5 | 0.51 | 0.05 |
| Exhaustion | 2 | 0.25 | 0.06 |
| Exhaustion | 3 | 0.36 | 0.06 |
| Exhaustion | 4 | 0.45 | 0.05 |
| Exhaustion | 5 | 0.38 | 0.06 |
| Loneliness | 2 | 0.1 | 0.07 |
| Loneliness | 3 | 0.21 | 0.06 |
| Loneliness | 4 | 0.43 | 0.05 |
| Loneliness | 5 | 0.35 | 0.06 |
| Cognition | 2 | 0.25 | 0.06 |
| Cognition | 3 | 0.29 | 0.06 |
| Cognition | 4 | 0.42 | 0.06 |
| Cognition | 5 | 0.5 | 0.05 |
| Anxiety | 2 | 0.17 | 0.07 |
| Anxiety | 3 | 0.27 | 0.06 |
| Anxiety | 4 | 0.34 | 0.06 |
| Anxiety | 5 | 0.37 | 0.06 |
| Sadness/Depression | 2 | 0.3 | 0.06 |
| Sadness/Depression | 3 | 0.34 | 0.06 |
| Sadness/Depression | 4 | 0.42 | 0.06 |
| Sadness/Depression | 5 | 0.43 | 0.06 |
| Control | 2 | 0.21 | 0.07 |
| Control | 3 | 0.21 | 0.07 |
| Control | 4 | 0.38 | 0.06 |
| Control | 5 | 0.42 | 0.05 |
| Pain | 2 | 0.31 | 0.06 |
| Pain | 3 | 0.3 | 0.06 |
| Pain | 4 | 0.38 | 0.06 |
| Pain | 5 | 0.42 | 0.06 |
| **GP sample** | | | |
| Mobility | 2 | 0.13 | 0.11 |
| Mobility | 3 | 0.42 | 0.10 |
| Mobility | 4 | 0.5 | 0.09 |
| Mobility | 5 | 0.55 | 0.08 |
| Daily activities | 2 | 0.24 | 0.11 |
| Daily activities | 3 | 0.42 | 0.10 |
| Daily activities | 4 | 0.47 | 0.09 |
| Daily activities | 5 | 0.51 | 0.09 |
| Exhaustion | 2 | 0.12 | 0.11 |
| Exhaustion | 3 | 0.47 | 0.09 |
| Exhaustion | 4 | 0.56 | 0.08 |
| Exhaustion | 5 | 0.5 | 0.09 |
| Loneliness | 2 | 0.086 | 0.12 |
| Loneliness | 3 | 0.23 | 0.11 |
| Loneliness | 4 | 0.55 | 0.08 |
| Loneliness | 5 | 0.55 | 0.11 |
| Cognition | 2 | 0.67 | 0.08 |
| Cognition | 3 | 0.53 | 0.09 |
| Cognition | 4 | 0.53 | 0.08 |
| Cognition | 5 | 0.52 | 0.08 |
| Anxiety | 2 | 0.22 | 0.11 |
| Anxiety | 3 | 0.27 | 0.11 |
| Anxiety | 4 | 0.29 | 0.11 |
| Anxiety | 5 | 0.35 | 0.10 |
| Sadness/Depression | 2 | 0.55 | 0.08 |
| Sadness/Depression | 3 | 0.49 | 0.09 |
| Sadness/Depression | 4 | 0.41 | 0.10 |
| Sadness/Depression | 5 | 0.46 | 0.09 |
| Control | 2 | 0.31 | 0.11 |
| Control | 3 | 0.24 | 0.11 |
| Control | 4 | 0.42 | 0.10 |
| Control | 5 | 0.41 | 0.10 |
| Pain | 2 | 0.42 | 0.10 |
| Pain | 3 | 0.31 | 0.11 |
| Pain | 4 | 0.25 | 0.11 |
| Pain | 5 | 0.35 | 0.10 |
| **Patient sample** | | | |
| Mobility | 2 | 0.13 | 0.08 |
| Mobility | 3 | 0.32 | 0.07 |
| Mobility | 4 | 0.45 | 0.07 |
| Mobility | 5 | 0.47 | 0.06 |
| Daily activities | 2 | 0.23 | 0.08 |
| Daily activities | 3 | 0.24 | 0.08 |
| Daily activities | 4 | 0.38 | 0.07 |
| Daily activities | 5 | 0.51 | 0.06 |
| Exhaustion | 2 | 0.35 | 0.07 |
| Exhaustion | 3 | 0.3 | 0.07 |
| Exhaustion | 4 | 0.39 | 0.07 |
| Exhaustion | 5 | 0.32 | 0.07 |
| Loneliness | 2 | 0.11 | 0.08 |
| Loneliness | 3 | 0.2 | 0.08 |
| Loneliness | 4 | 0.39 | 0.07 |
| Loneliness | 5 | 0.34 | 0.07 |
| Cognition | 2 | 0.1 | 0.08 |
| Cognition | 3 | 0.17 | 0.08 |
| Cognition | 4 | 0.38 | 0.07 |
| Cognition | 5 | 0.48 | 0.06 |
| Anxiety | 2 | 0.13 | 0.08 |
| Anxiety | 3 | 0.25 | 0.08 |
| Anxiety | 4 | 0.38 | 0.07 |
| Anxiety | 5 | 0.38 | 0.08 |
| Sadness/Depression | 2 | 0.19 | 0.08 |
| Sadness/Depression | 3 | 0.26 | 0.08 |
| Sadness/Depression | 4 | 0.43 | 0.07 |
| Sadness/Depression | 5 | 0.42 | 0.07 |
| Control | 2 | 0.17 | 0.08 |
| Control | 3 | 0.19 | 0.08 |
| Control | 4 | 0.35 | 0.07 |
| Control | 5 | 0.43 | 0.07 |
| Pain | 2 | 0.27 | 0.08 |
| Pain | 3 | 0.29 | 0.07 |
| Pain | 4 | 0.4 | 0.07 |
| Pain | 5 | 0.44 | 0.07 |

**Online Resource 4. Regression analysis of cumulative difference in utility decrements: Main effects (model 1) and interaction effects (model 2) of age, gender, and patient sample**

|  | **Model 1 coefficient** | **p-value** | **Model 2 coefficient** | **p-value** |
| --- | --- | --- | --- | --- |
| **Age: 50-64 years^a^** | 0.15 | 0.01* | 0.07 | 0.38 |
| **Age: 65+ years^a^** | 0.07 | 0.19 | 0.04 | 0.69 |
| **Sample: Patient^b^** | -0.04 | 0.43 | -0.11 | 0.17 |
| **Gender: Male^c^** | -0.08 | 0.05 | -0.08 | 0.046* |
| **Interaction: Age: 50-64 years^a^ Sample: Patient^b^** |  |  | 0.16 | 0.17 |
| **Interaction: Age: 65+ years^a^ Sample: Patient^b^** |  |  | 0.08 | 0.52 |
| **Number of observations** | 219 |  | 219 |  |
| **Adjusted R-squared** | 0.03 |  | 0.03 |  |

*p<0.05

^a^Reference group: 18-49 years, ^b^Reference group: general population sample, ^c^Reference group: Female
